# Additive Interaction Between Onabotulinumtoxin-A and Erenumab in Patients With Refractory Migraine

**DOI:** 10.3389/fneur.2021.656294

**Published:** 2021-04-08

**Authors:** Marcello Silvestro, Alessandro Tessitore, Fabrizio Scotto di Clemente, Giorgia Battista, Gioacchino Tedeschi, Antonio Russo

**Affiliations:** Department of Advanced Medical and Surgical Sciences, Headache Centre, University of Campania “Luigi Vanvitelli, ” Naples, Italy

**Keywords:** monoclonal antibodies, erenumab, onabotulinumtoxin-A, refractory migraine, migraine

## Abstract

In the last decade, notable progresses have been observed in chronic migraine preventive treatments. According to the European Headache Federation and national provisions, onabotulinumtoxin-A (BTX-A) and monoclonal antibodies acting on the pathway of calcitonin gene–related peptide (CGRP-mAbs) should not be administered in combination due to supposed superimposable mechanism of action and high costs. On the other hand, preclinical observations demonstrated that these therapeutic classes, although operating directly or indirectly on the CGRP pathway, act on different fibers. Specifically, the CGRP-mAbs prevent the activation of the Aδ-fibers, whereas BTX-A acts on C-fibers. Therefore, it can be argued that a combined therapy may provide an additive or synergistic effect on the trigeminal nociceptive pathway. In the present study, we report a case series of 10 patients with chronic migraine who experienced significant benefits with the combination of both erenumab and BTX-A compared to each therapeutic strategy alone. A reduction in frequency and intensity of headache attacks (although not statistically significant probably due to the low sample size) was observed in migraine patients treated with a combined therapy with BTX-A and erenumab compared to both BTX-A and erenumab alone. Moreover, the combined therapy with BTX-A and erenumab resulted in a statistically significant reduction in the symptomatic drug intake and in migraine-related disability probably related to a reduced necessity or also to a better responsiveness to rescue treatments. Present data suggest a remodulation of current provisions depriving patients of an effective therapeutic strategy in peculiar migraine endophenotypes.

## Introduction

In the last decade, after a period of substantial stagnation, notable progresses have been observed in chronic migraine preventive treatments because of the advent of new, specific therapeutic strategies characterized by an equal or even higher efficacy in comparison with previous standards of care but, overall, by better tolerability profiles (1). We refer in particular to onabotulinumtoxin-A (BTX-A) and monoclonal antibodies targeting calcitonin gene–related peptide (CGRP) peptide or its receptor (CGRP-mAbs), both showing to be effective in migraine preventive treatment in randomized, controlled, double-blind trials as well as in real-world experiences (2–4). A recent consensus suggested the CGRP-mAbs administration in chronic migraine patients only in case of previous failure of BTX-A therapy and, overall, never in combination owing to almost superimposable mechanisms of action against CGRP pathway and high cost of each treatment (5). On the other hand, recent preclinical and clinical observations demonstrated that these two therapeutic classes, although operating directly or indirectly on the same CGRP pathway, seem to act on different fibers (6–8). Specifically, the CGRP-mAbs may prevent the activation of the Aδ-fibers, whereas BTX-A can act on C-fibers. Therefore, it can be argued that a combined therapy may provide an additive or synergistic effect on the trigeminal nociceptive pathway. We report a case series of chronic migraine patients who reported a significant benefit with the combination of both erenumab and BTX-A compared to each therapeutic strategy alone.

## Methods

### Population

We refer the cases of 10 patients meeting criteria for both chronic migraine and medication-overuse headache according to the International Headache Society criteria (9) referring to the Headache Center of the Department of Neurology at the University of Campania “Luigi Vanvitelli” between August 2019 and September 2020. These patients, aged between 18 and 65 years, failed at least four or more oral preventive medication classes (propranolol or metoprolol, topiramate, flunarizine, valproate, amitriptyline, or candesartan) due to lack of efficacy or intolerable side effects. For this reason, all patients previously underwent BTX-A therapy (according to the PREEMPT protocol with “follow-the-pain” approach) for at least 9 months (e.g., three administrations of 185 UI of BTX-A) (2, 3). In the course of BTX-A treatment, we observed a >30– <50% reduction in monthly headache days (MHDs) and/or severity of headache during attacks compared to the baseline. Because the migraine burden decrease was considered not satisfactory based on both the headache-related disability [evaluated by Migraine Disability Assessment (MIDAS)] and the symptomatic drug intake, according to patients' decision, BTX-A treatment was discontinued, whereupon after several months from the latest BTX-A administration (from 6 to 12 months), due to headaches resurgence, erenumab 140 mg monthly administration was started and maintained for at least 6 months. After six erenumab 140 mg monthly administrations (at the 7th month), patients reported a >30– <50% reduction in MHDs and/or severity of headache during attacks compared to the baseline, not dissimilar to what was observed during the previous BTX-A treatment. Nevertheless, migraine burden decrease was again reported not satisfactory by the patients, once again considering the headache-related disability (by MIDAS) and the symptomatic drug intake. In other terms, our patients fulfilled the European Headache Federation criteria for “refractory migraine” (10). Although a >30% reduction in headache days can be considered satisfactory, it has been recently argued that with the use of percent reduction, patients may be considered responders but still have a relevant number of debilitating days with headache. Indeed, to overcome this problem, the European Headache Federation (EHF) Expert Consensus Group opted to use eight headache days per month as cutoff values. Eight days per month were chosen to consider evidence indicating that moderate disability starts after 4 migraine days per month (10). Therefore, to achieve a putative additive or synergic interaction, a combined treatment with BTX-A (185 UI quarterly administration) and erenumab (140 mg monthly administration) was started. Then, after 6 months of combined treatment, we evaluated MHDs, severity of headache during attacks, symptomatic drug intake per month, and migraine disability (see Table 1 for further information). We were able to administer both BTX-A and erenumab as the first is provided by the Italian national health system, whereas the second has been administered in migraine patients by means of a special modality of acquisition where a symbolic cost of erenumab was borne entirely by the hospital in order to guarantee the accessibility to the drug until the conclusion of the erenumab national price negotiation process. Each patient gave a free, informed consent for publication of the clinical data.

**Table 1:** Descriptive statistics of 10 patients characteristics and evolution of clinical parameters of disease severity.

		**BNT_A vs. baseline**	**Erenumab vs. baseline**	**Erenumab vs. BNT_A**	**Combined therapy vs. baseline**	**Combined therapy vs. BNT_A**	**Combined therapy vs. erenumab**
Female n (%)	7 (70%)	–	–	–	–	–	–
Age (Mean ± SD)	47.33 ± 9.30	–	–	–	–	–	–
Headache history (Mean ± SD)	30.55 ± 11.98	–	–	–	–	–	–
Baseline frequency of headache attacks (Median ± IQR)	25 ± 13	–	–	–	–	–	–
Baseline headache pain intensity (Median NRS ± IQR)	9 ± 1	–	–	–	–	–	–
Baseline pain killers intake (Median ± IQR)	24 ± 15	–	–	–	–	–	–
Baseline migraine related disability by MIDAS (Median ± IQR)	125 ± 56	–	–	–	–	–	–
Frequency of headache attacks after three BNT_A administrations (Median ± IQR)	17 ± 7	**0.012**	–	–	–	–	–
Headache pain intensity after three BNT_A administrations (Median NRS ± IQR)	7 ± 1	**0.010**	–	–	–	–	–
Pain killers intake after three BNT_A administrations (Median ± IQR)	15 ± 10	**0.008**	–	–	–	–	–
Migraine related disability by MIDAS after three BNT_A administrations (Median ± IQR)	102 ± 58	**0.008**	–	–	–	–	–
Frequency of headache attacks after six Erenumab administrations (Median ± IQR)	13 ± 5	–	**0.008**	0.371	–	–	–
Headache pain intensity after six Erenumab administrations (Median NRS ± IQR)	8 ± 1	–	**0.016**	0.060	–	–	–
Pain killers intake after six Erenumab administrations (Median ± IQR)	13 ± 8	–	**0.007**	0.159	–	–	–
Migraine related disability by MIDAS after six Erenumab administrations (Median ± IQR)	72 ± 53	–	**0.008**	0.482	–	–	–
Frequency of headache attacks after 6 months of combined therapy (Median ± IQR)	10 ± 3	–	–	–	**0.008**	**0.007**	**0.008**
Headache pain intensity after 6 months of combined therapy (Median NRS ± IQR)	7 ± 1	–	–	–	**0.008**	**0.012**	**0.008**
Pain killers intake after 6 months of combined therapy (Median ± IQR)	7 ± 5	–	–	–	**0.008**	**0.008**	**0.007**
Migraine related disability by MIDAS after 6 months of combined therapy (Median ± IQR)	24 ± 12	–	–	–	**0.008**	**0.006**	**0.008**

*SD, standard deviation; NRS, numerical rating scale; BNT_A, Onabotulinumtoxin-A; MIDAS, migraine disability assessment; IQR, interquartile range*.

*Bold values means statistically significant (p < 0.05)*.

### Statistical Analysis

Continuous data were reported as mean ± standard deviation, and scale scores were reported as median and interquartile range. We used *t*-test to compare continuous variables and Wilcoxon–Mann–Whitney to compare medians. Statistical significance was set at *p* < 0.05. Because of the observational design of the study, we did not plan a sample size calculation. All analyses were performed using STATA version 14 (StataCorp, College Station, TX, USA).

## Results

**Figure 1:**
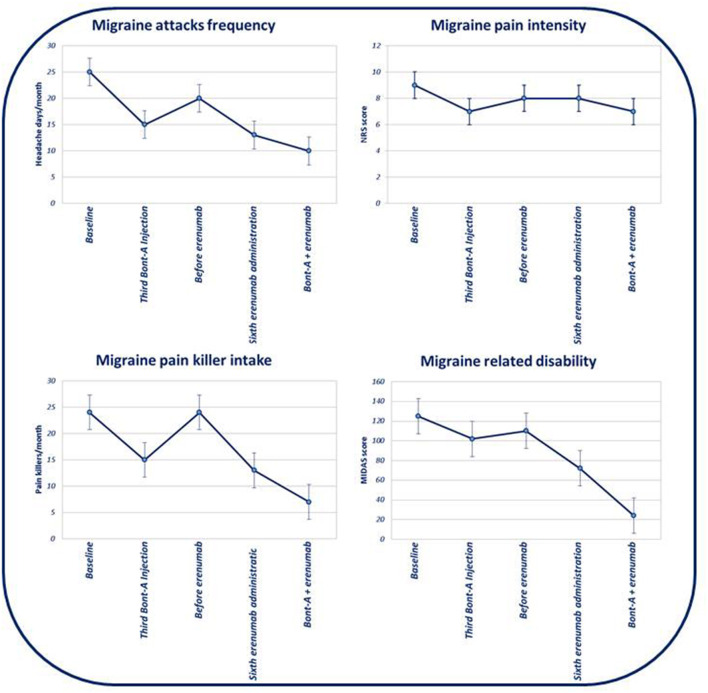
Evolution of monthly headache days' frequency, headache attacks' pain intensity, pain killer intake, and evolution of migraine-related disability (based on MIDAS) in the course of subsequent therapies.

**Figure 2:**
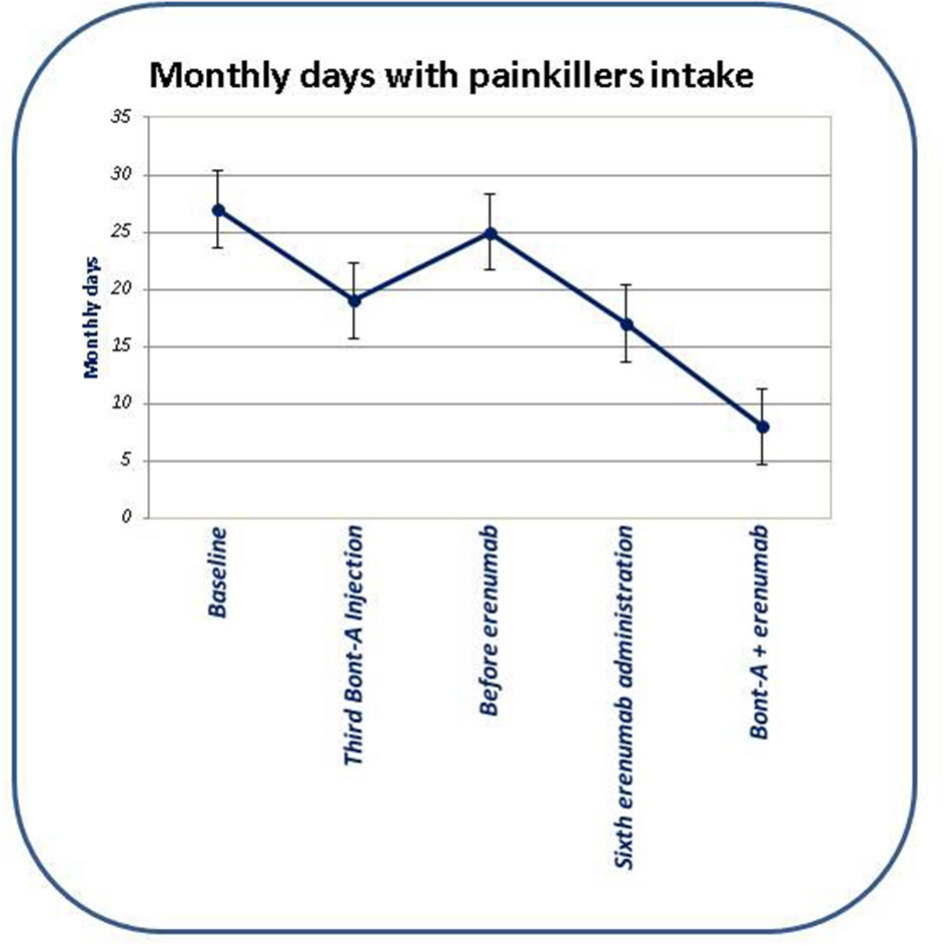
Evolution of monthly days with painkillers intake in the course of subsequent therapies.

After three BTX-A185 UI quarterly administrations combined with six erenumab 140 mg monthly administrations, we observed a statistically significant reduction of MHDs (*p* < 0.01), intensity of headache during attacks (*p* < 0.01), and symptomatic drug intake per month (*p* < 0.01), as well as migraine disability (by using MIDAS) (*p* < 0.01), compared to the baseline. Moreover, comparing the combined therapy (e.g., BTX-A and erenumab) with both BTX-A and erenumab treatments alone, we demonstrated a reduction of MHDs and severity of headache during attacks (*p* < 0.01) and a concomitant statistically significant reduction of symptomatic drug intake per month (*p* < 0.01) and, overall, a statistically significant improvement of migraine disability (*p* < 0.01) (evaluated with MIDAS) (see Table 1 and Figures 1, 2 for further information). During BTX-A treatment, 30% of patients reported pain in the injection sites for no more than 48 h. We found 20% of patients reporting mild constipation in the course of treatment with erenumab alone. During combined therapy, we did not observe an increased percentage of side effects. There were no serious AEs, and no patient discontinued treatment due to adverse events.

## Discussion

In the last decade, notable progresses have been observed in chronic migraine preventive approaches because of the advent of new therapeutic strategies such as BTX-A and CGRP-mAbs characterized by high effectiveness and increased tolerability and safety profile compared to previous antimigraine preventive oral medications (1). BTX-A has represented, until the approval of CGRP-mAbs, the only therapy specifically approved as preventive treatment for chronic migraine. Randomized, double-blind, placebo-controlled trials, reported a >50% reduction from the baseline in MHDs in the 47.1% of patients compared to 35.1% of the placebo group (2, 3). Furthermore, real-life experiences have confirmed the high effectiveness and tolerability of BTX-A treatment (11). CGRP is a neuropeptide primarily expressed in the central and peripheral nervous system playing a pivotal role in migraine genesis and maintenance. BTX-A is supposed to block peripheral sensitization through the inhibition of pain-mediating peptides release, especially CGRP, from peripheral nociceptive neurons where the reversal of peripheral sensitization may indirectly lead to central sensitization reversion (12, 13). CGRP-mAbs represent the first selective therapeutic approach specific for migraine prevention. Among these, erenumab is a fully human monoclonal antibody selectively targeting and blocking the CGRP receptor. Randomized investigations reported a >50% reduction from the baseline in monthly migraine days (MMDs) in the 34.8 and 38.5% of chronic migraine patients treated with, respectively, 70 and 140 mg monthly erenumab administration compared to 15.3% of the placebo group (14). Real-life observations have recently demonstrated significantly better results with a percentage of responders (>50% reduction of MMDs), ranging between the 48 and 53% after the third erenumab administration (15, 16). A recent consensus of the European Headache Federation and national provisions (e.g., Italian and German medicine agencies) stated that BTX-A and CGRP-mAbs should not be administered in combination due to both a supposed superimposable mechanism of action and the high cost of each therapy (17). However, recent observations demonstrated that CGRP-mAbs prevent the activation of the Aδ-fibers but not C-fibers, whereas BTX-A prevents the activation of the C-fibers but not Aδ-fibers (7, 8). Consequently, one can argue that CGRP-mAbs may be effective in migraine patients with predominant involvement of the Aδ-fibers and high-threshold neurons, whereas migraine patients non-responsive to CGRP-mAbs could be characterized by a higher involvement of the C-fibers and/or different central trigeminovascular neurons (18, 19). More recently, to provide rational clinical evidences of a combined therapy acting on the trigeminal nociceptive pathway (e.g., both Aδ-fibers and C-fibers), the synergistic effect of BTX-A and erenumab has been evaluated in a cohort of chronic migraine patients (11, 20). In the study, a retrospective chart review of 78 patients investigated the therapeutic advantage in the MHDs and MMDs provided by the addition of erenumab to patients with chronic migraine who were receiving BTX-A. The authors reported a significant decrease in MHDs and MMDs in the course of the combined treatment with a specific reduction of 8.1 MHDs and 7.4 MMDs in a total of 90 days. Similarly, during the 2020 American Headache Society Meeting, Cohen reported, by a retrospective chart review, that the add-on of CGRP-mAbs therapy (e.g., erenumab, galcanezumab, or fremanezumab) to BTX-A was associated with an additional decrease of MMDs compared with BTX-A alone in 153 chronic migraine patients with a therapeutic gain of 5.6 days. However, the aforementioned observations lack comparison groups (i.e., chronic migraine patients receiving CGRP-mAbs but not BTX-A treatment), and therefore, the possibility that clinical outcomes improvement was carried over by CGRP-mAbs therapeutic effect alone (more than to the combination of the two strategies) cannot be excluded. In the present study, a reduction in frequency and intensity of headache attacks was observed in migraine patients treated with a combined therapy with BTX-A and erenumab compared to both BTX-A and erenumab alone. Moreover, the combined therapy with BTX-A and erenumab resulted in a statistically significant reduction in the symptomatic drug intake and in migraine-related disability probably related to a reduced necessity or also to a better responsiveness to rescue treatments. It is a common experience for clinicians dealing with chronic migraine to face patients who only partially respond to a monotherapy using preventive medication. If meaningful effectiveness is not obtained, the addition of a second medication with an additive or synergistic effect can be potentially attractive. Indeed, headache specialists have argued in favor of polytherapy strategies to deal with resistant migraine (21). Even more, combined therapy with erenumab and BTX-A, considering efficacy and safety profiles characterizing these molecules, could represent an optimal therapeutic strategy to deal with otherwise refractory migraine patients (10). The main limitation of the present observation is the low sample size, which could justify the statistically significant reduction in migraine-related disability and pain killer intake. In conclusion, our results represent, despite the limitation due to the low sample size, the concept that a combined therapy may provide an additive or synergistic effect on the trigeminal nociceptive pathway, as has been recently argued on the basis of preclinical insights. However, Italian medicine agency provisions, due to the absence of reliable and reproducible data about the additive interaction of BTX-A and erenumab, made us discontinue our experience using the combined therapy in patients with refractory migraine. Nevertheless, we are aware that translating these acquisitions in clinical practice could be very arduous, overall because the combined strategy with BTX-A and erenumab is very expensive and not easily bearable by the national health systems, also considering the high prevalence of migraine in the general population. However, we hope that the present data, showing an additive therapeutic effect of BTX-A and erenumab, can be taken into account to suggest a remodulation of current provisions that deprive clinicians of an effective therapeutic strategy in migraine patients with inadequate pain relief, despite several acute and preventive treatments, experiencing higher burden, disability, and despair.

## Data Availability Statement

The raw data supporting the conclusions of this article will be made available by the authors, without undue reservation.

## Ethics Statement

The studies involving human participants were reviewed and approved by Ethical Committee of the University of Campania Luigi Vanvitelli. The patients/participants provided their written informed consent to participate in this study.

## Author Contributions

MS: data analysis, literature review, results interpretation, manuscript drafting, and revision. AT: data analysis, results interpretation, and manuscript revision. FS: literature review, results interpretation, and manuscript revision. GB and GT: results interpretation and manuscript revision. AR: data analysis, results interpretation, manuscript drafting, and revision. All authors contributed to the article and approved the submitted version.

## Conflict of Interest

MS has received speaker honoraria from Novartis and Lilly. AT has received speaker honoraria from Novartis, Schwarz Pharma/UCB, Lundbeck, Abbvie, and Glaxo. GT has received speaker honoraria from Sanofi-Aventis, Merck Serono, Bayer Schering Pharma, Novartis, Biogen-Dompe' AG, Teva and Lilly; has received funding for travel from Bayer Schering Pharma, Biogen-Dompe' AG, Merck Serono, Novartis and Sanofi Aventis; and serves as an associate editor of Neurological Sciences. AR has received speaker honoraria from Allergan, Lilly, Novartis and Teva and serves as an associate editor of Frontiers in Neurology (Headache Medicine and Facial Pain session). The remaining authors declare that the research was conducted in the absence of any commercial or financial relationships that could be construed as a potential conflict of interest.
